# Compositional features analysis by machine learning in genome represents linear adaptation of monkeypox virus

**DOI:** 10.3389/fgene.2024.1361952

**Published:** 2024-03-01

**Authors:** Sen Zhang, Ya-Dan Li, Yu-Rong Cai, Xiao-Ping Kang, Ye Feng, Yu-Chang Li, Yue-Hong Chen, Jing Li, Li-Li Bao, Tao Jiang

**Affiliations:** ^1^ State Key Laboratory of Pathogen and Biosecurity, Beijing Institute of Microbiology and Epidemiology, Academy of Military Medical Sciences, Beijing, China; ^2^ College of Basic Medical Sciences, Anhui Medical University, Hefei, China; ^3^ College of the First Clinical Medical, Inner Mongolia Medical University, Hohhot, China; ^4^ College of Basic Medical Sciences, Inner Mongolia Medical University, Hohhot, China

**Keywords:** monkeypox viruses, machine learning, linear adaptation, open reading frame clusters, dinucleotide composition representation (DCR)

## Abstract

**Introduction:** The global headlines have been dominated by the sudden and widespread outbreak of monkeypox, a rare and endemic zoonotic disease caused by the monkeypox virus (MPXV). Genomic composition based machine learning (ML) methods have recently shown promise in identifying host adaptability and evolutionary patterns of virus. Our study aimed to analyze the genomic characteristics and evolutionary patterns of MPXV using ML methods.

**Methods:** The open reading frame (ORF) regions of full-length MPXV genomes were filtered and 165 ORFs were selected as clusters with the highest homology. Unsupervised machine learning methods of t-distributed stochastic neighbor embedding (t-SNE), Principal Component Analysis (PCA), and hierarchical clustering were performed to observe the DCR characteristics of the selected ORF clusters.

**Results:** The results showed that MPXV sequences post-2022 showed an obvious linear adaptive evolution, indicating that it has become more adapted to the human host after accumulating mutations. For further accurate analysis, the ORF regions with larger variations were filtered out based on the ranking of homology difference to narrow down the key ORF clusters, which drew the same conclusion of linear adaptability. Then key differential protein structures were predicted by AlphaFold 2, which meant that difference in main domains might be one of the internal reasons for linear adaptive evolution.

**Discussion:** Understanding the process of linear adaptation is critical in the constant evolutionary struggle between viruses and their hosts, playing a significant role in crafting effective measures to tackle viral diseases. Therefore, the present study provides valuable insights into the evolutionary patterns of the MPXV in 2022 from the perspective of genomic composition characteristics analysis through ML methods.

## 1 Introduction

The current human monkeypox outbreak since May 2022 has quickly spread to 116 countries, caused more than 90,000 confirmed cases and 167 deaths as of October 2023 (WHO, 2023), showing no self-limiting with taking steps at the global level. The causative agent of monkeypox virus (MPXV) belongs to the Orthopoxvirus genus, which also comprises variola virus (causing smallpox), vaccinia virus, and cowpox virus (Brown and Leggat, 2016; Mitjà and Ogoina et al., 2023). As a zoonotic virus, MPXV is probably harbored in natural mammals such as nonhuman primates, rodents, squirrels (McCollum and Damon, 2014; Brown and Leggat, 2016; Durski et al., 2018; Bunge et al., 2022), sporadically infects human on the occasion of close contact with the animal reservoir/reservoirs. MPXV infection cause a smallpox-like disease in humans, additionally with the distinguishing lymphadenopathy (Durski et al., 2018). Human monkeypox was recorded to infect human primarily in DR Congo in 1970 (Ladnyj et al., 1972), then in West Africa area (Di Giulio and Eckburg, 2004) and to sporadically outbroke in central and west Africa (Di Giulio and Eckburg, 2004; Gong and Wang, 2022). There are two distinct monkeypox virus clades of the Congo Basin clade and the west African clade, respectively responsible for the outbreaks in the two areas (Brown and Leggat, 2016; Karagoz et al., 2023), and only the former clade was initially documented to be transmissible in human population and high pathogenic (Reynolds et al., 2007; Karagoz et al., 2023). Worryingly, the current outbroken MPXV is also human to human transmissible, and its origin and genomic characterization have not been concluded, though the analysis based on the sequencing data in early outbreak stage indicated the current prevalent MPXV the belonged to the west Africa clade (Isidro et al., 2022).

MPXV is an enveloped double-stranded DNA virus, which is also one of the largest and the most complex among all known human and animal viruses (Canessa, 2022). Their genome contains up to 197 open reading frames (ORFs), encoding more than 200 different proteins (Canessa, 2022; Sereewit et al., 2022). Unlike influenza virus (Deng et al., 2017), SARS-Cov-2 (D and S et al., 2023) and other RNA viruses, most of the protein-coding genes are highly conserved among the members of Orthopoxvirus genus (Gershon et al., 1989; Gillard et al., 1989). Due to the complexity and stability of MPXV genome, research on its genomic characteristics is somewhat challenging. The annual mutation rate of MPXV genome before 2022 is slow with 1-2 substitutions per year (Firth et al., 2010). Studies have shown that there was an explosive single-nucleotide polymorphisms (SNP) mutation in 2022 MPXV (about 50) compared with the previous sequences, which might be one of the important reasons for the sudden outbreak of monkeypox (Isidro et al., 2022). However, current research still lacks a comprehensive analysis of the overall characteristics of the full-length genome of MPXV, thus it may not be able to fully explain the evolutionary patterns and directions of MPXV.

Sequence composition characteristics of nucleic acids and proteins are significantly related to biological evolution (Liam and Fowler, 2021; Shuai et al., 2022). The viral genome features representing the virus evolution patterns information can be transformed into language representation that can be learned by artificial intelligence methods (Brian et al., 2021). Machine learning (ML) has performed well to predict virus evolution, viral host adaptation or viral pathogenicity based on the nucleotide or amino acids composition (Simon et al., 2018; Jing et al., 2020; Jing et al., 2022). Dinucleotide composition representation (DCR) characterization method has been proved well in analysis of viral evolution trends and host adaptation prediction in SARS-Cov-2 (Jing et al., 2022), bat coronaviruses (Jing et al., 2023) and swine coronaviruses (Daniele et al., 2023). Machine learning methods based on DCR characteristics can distinguish small differences in viral genomes, thus making scientific predictions about the further evolutionary trends of viral genomes. Therefor, machine learning methods are expected to analyze the full-length genome composition characteristics of MPXV, in order to provide a certain degree of explanation for the evolutionary trends of MPXV post 2022.

The present study aimed to infer the genomic characteristics of 2022 MPXV using machine learning methods, in comparison to earlier virus data from the same West African clade, or virus genome sequences from the Congo Basin clade. We have filtered the ORF regions of all MPXV genomes and selected 165 ORFs as clusters with the highest homology. Unsupervised machine learning methods of t-distributed stochastic neighbor embedding (t-SNE), Principal Component Analysis (PCA), and hierarchical clustering were performed to observe the DCR characteristics in the selected ORF clusters. Then, in order to better explain their evolutionary patterns, the ORF regions with larger variations were filtered out based on the ranking of DCR characteristics to narrow down the key ORF clusters for further unsupervised machine learning. Our research provides valuable insights into the evolutionary patterns of the MPXV in 2022.

## 2 Materials and methods

### 2.1 Data processing and ORFs screening of MPXV

More than 7,000 full genome sequences of MPXV were downloaded from the National Center of Biotechnology Information (NCBI) database (https://www.ncbi.nlm.nih.gov/nuccore) up to 3 November 2023. An amount of 6,822 high-quality sequences were selected, with 383 were before 2022 and 6439 post 2022. Two reference MPXV sequences (NC_003310.1 and NC_063383.1), which were also the designated reference sequences in NCBI, were selected to manually filter the ORF regions as templates. By comparing the full-length sequences of all cleaned MPXV with template ORF sequences, and calculating the levenshtein distance (LD) value (threshold less than 0.05), the ORF regions of all sequences could be obtained. The calculation formula of LD is as follows:

Then through homology analysis, 165 ORF regions with highest homology were selected as clusters.

### 2.2 Genomic compositional characteristics parsing of MPXV ORF clusters

A nucleotide counting script of python was utilized for genome sequence decomposition (Jing et al., 2022). The frequency of compositional characteristics, 1536 dinucleotide composition representations (DCRs), was determined for each MPXV sample’s ORF clusters sequence using the following formula: ‘count’ represents the quantity, and ‘seq_len’ represents the total length of the selected gene sequence.

### 2.3 Reduction, visualization, and clustering of DCR characteristics of MPXV ORF clusters

Dimension reduction techniques such as Principal Component Analysis (PCA) and t-Distributed Stochastic Neighbor Embedding (t-SNE) were utilized to visualize data distribution and clustering for the full-dimensional features of 1,536 DCRs for MPXV ORF clusters. The PCA and t-SNE procedures were carried out using sklearn.decomposition.PCA (Jolliffe and Cadima, 2016) and sklearn.manifold.TSNE (https://scikit-learn.org/stable/about.html#citing-scikit-learn), respectively. The Python Seaborn package was used to plot two main components (PCA1 and PCA2, or t-SNE1 and t-SNE2) with a collection date label for each data point. An unsupervised machine learning approach based on hierarchical clustering was then employed to observe the clustering and homology of MPXV with various collection date labels, using the full-dimensional features of DCR compositional characteristics. Euclidean distance was used as a hierarchical clustering scalar, and the sns.clustermap package was utilized to perform hierarchical clustering. Additionally, to address the biased sample number and reduce the impact of sample differences between MPXV with the two collection date labels (before and post 2022) on machine learning, random down- and up-sampling were carried out using the imblearn.over_sampling.SMOTE package prior to dimension reduction and visualization.

### 2.4 Phylogenetic analysis of MPXV ORF clusters

In order to explore the phylogenetic relationship of the MPXV samples, phylogenetic trees were constructed on the basis of ORF clusters. The DNA sequences of all randomly sampled MPXV with known collection information were first aligned by MAFFT (Katoh et al., 2002), and maximum likelihood trees were constructed using RAxML v8.2.12 (Stamatakis, 2014) with 100 bootstrap iterations and other variables set to default. Phylogenetic trees were visualized using iTol (Letunic and Bork, 2016).

### 2.5 Protein structure prediction by AlphaFold 2

Protein structure prediction begins with the use of AlphaFold2 for prediction. The brief process is as follows: (1) Open and run the terminal in the Ubuntu system, activate the AlphaFold environment with “conda activate alphafold”; (2) Run the AlphaFold prediction model using the command “python/home/inspur/git_package/alphafold-main/docker/run_docker.py—fasta paths = /home/inspur/git_package/alphafold-main/MPXV_protein.fasta—max_template_data = 2020-05–14"; (3) Visualize the predicted results using PyMOL, open PyMOL with "/home/inspur/pymol/pymol”, then use the file toolbar to open the ranked0_.pdb file (the top-ranked predicted structure file) for visualizing the structure.

## 3 Results

### 3.1 Workflow of linear adaptive evolution analysis of MPXV

**FIGURE 1:**
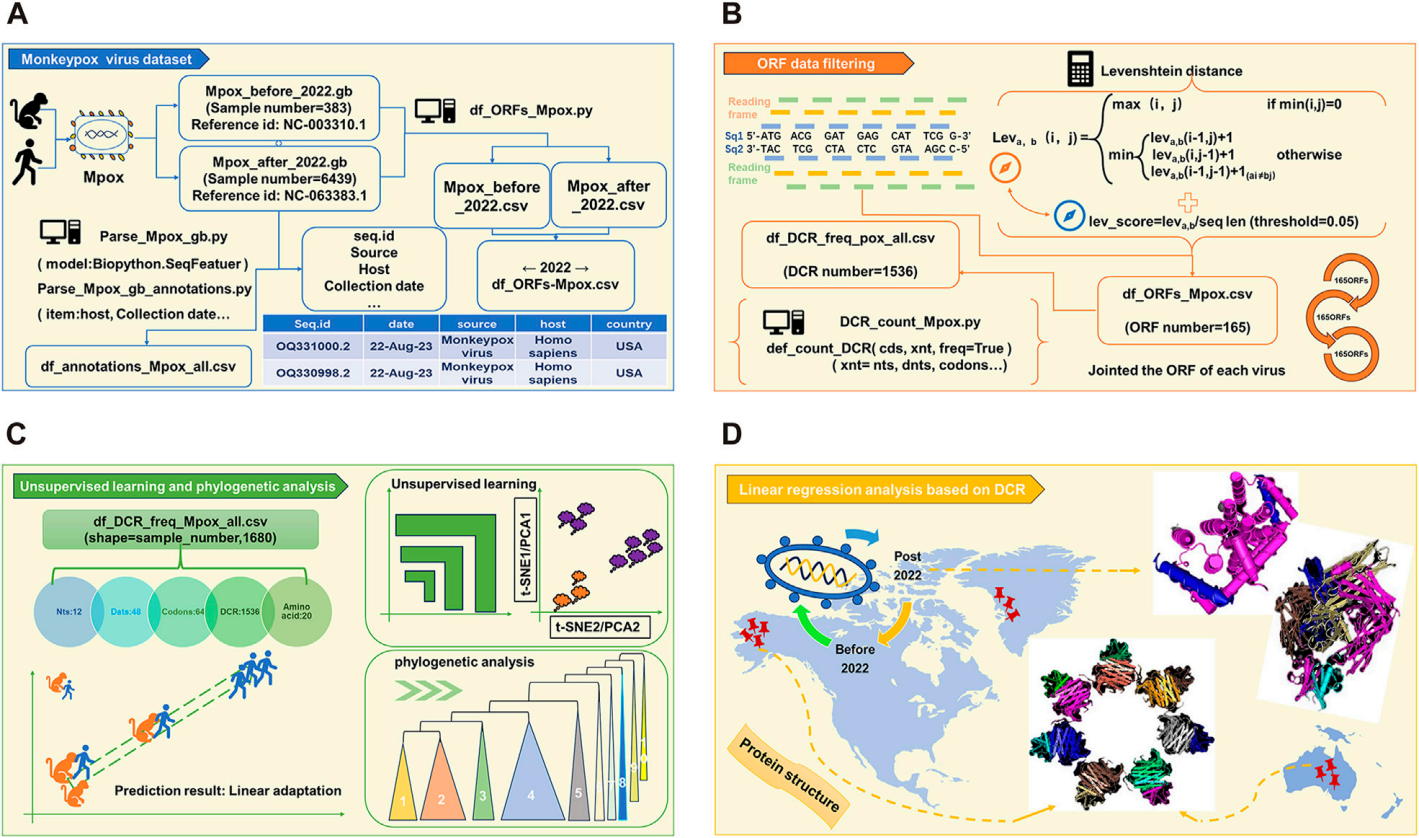
Workflow of linear adaptive evolution analysis of MPXV. The workflow was designed with four parts. **(A)** Download, cleaning and annotation of genomic sequence of MPXV. **(B)** Algorithm for filtering ORF data and the resulting output. **(C)** Schematic diagram of unsupervised learning and phylogenetic analysis. **(D)** Structure prediction of key mutant proteins by AlphaFold 2.

As the schematic diagram (Figure 1) shows, full length of all MPXV genome sequences were downloaded and cleaned first. The duplicate and incomplete sequences were removed, and the rest were classified according those uploaded time (before and post 2022). A total of 383 sequences before 2022 and 6439 sequences post 2022 were obtained, which were further annotated (Figure 1A). Then, two reference MPXV sequences (NC_003310.1 & NC_063383.1) were selected to manually filter the ORF regions as templates for following analysis. By comparing the full-length sequences of all cleaned MPXV with template ORF sequences, and calculating the levenshtein distance (LD) value (threshold less than 0.05), the ORF regions of all sequences could be obtained. Through homology analysis, 165 ORF regions with highest homology were selected as clusters for genome composition characteristics analysis (Figure 1B). The unsupervised projection methods of t-distributed stochastic neighbor embedding (t-SNE) and Principal Component Analysis (PCA) were utilized to learn the separation and linear adaptation of MPXV (Figure 1C). Finally, 30 ORF regions with greatest difference in DCR characteristics were selected for further analysis, and the protein structures with significant difference were predicted by AlphaFold2 (Figure 1D).

### 3.2 Unsupervised learning of highly homologous ORF clusters of MPXV

**FIGURE 2:**
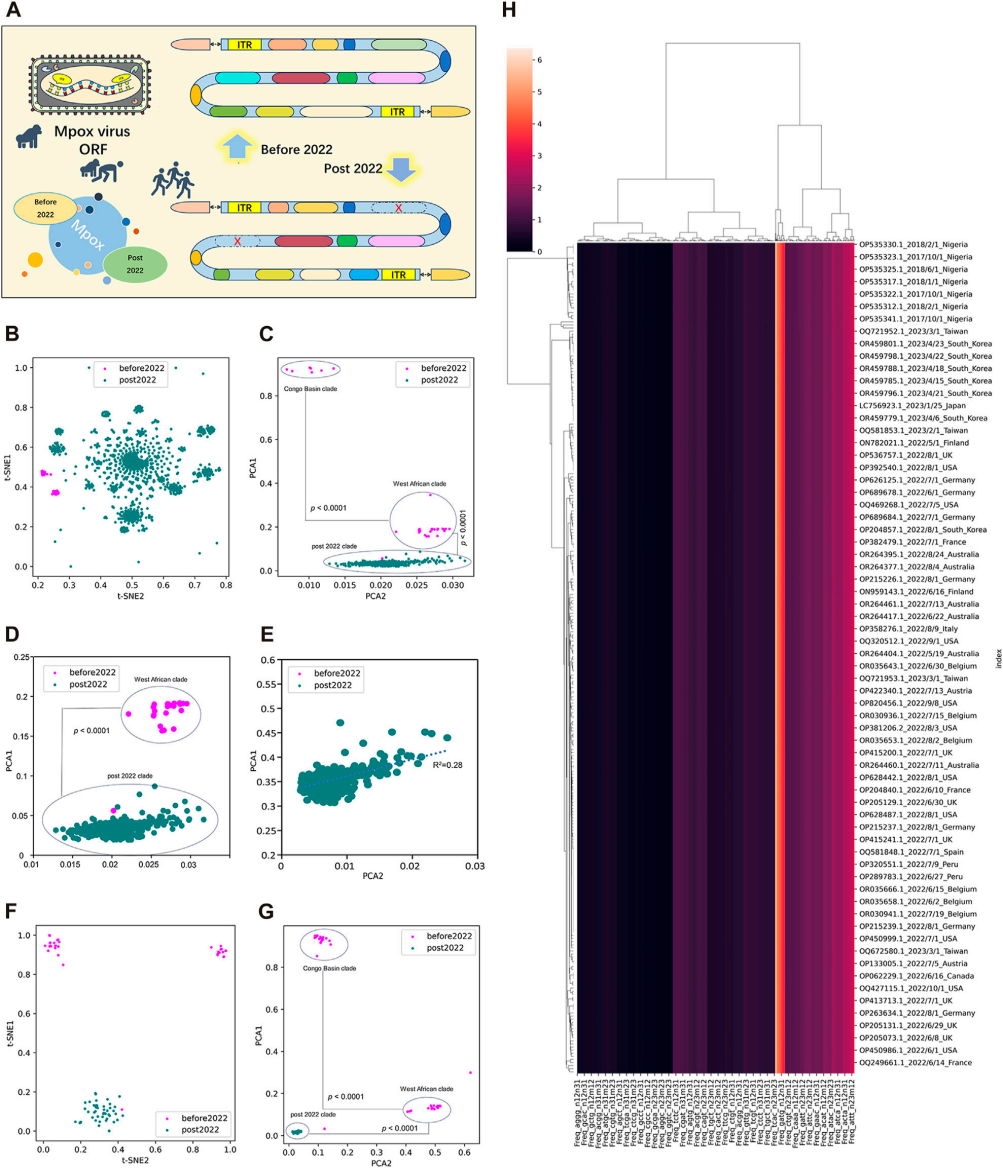
Unsupervised learning of highly homologous ORF clusters of MPXV. **(A)** ORF regions of all MPXV sequences were separated and screened according to homology analysis with reference sequences (NC_003310.1 and NC_063383.1). A total of 165 ORF regions were selected to form ORF clusters. **(B, C)** Visualization of DCR characteristics reduced with t-SNE **(B)** and PCA **(C)** of 165 ORF clusters from each MPXV sequence. **(D, E)** Visualization of DCR characteristics reduced with PCA of 165 ORF clusters from MPXV sequences except for the Congo Basin clade **(D)** and only the post 2022 MPXV clade **(E)**. **(F, G)** Visualization of DCR characteristics reduced with t-SNE **(D)** and PCA **(E)** of 165 ORF clusters from each randomly sampled MPXV sequence. **(H)** Hierarchical clustering of DCR characteristics of 165 ORF clusters from each randomly sampled MPXV sequence. Statistical significance in the PCA value difference between two neighboring clades is indicated, respectively, according to an unpaired, nonparametric Mann-Whitney test.

To better evaluate the adaption and evolution trends of MPXV, the ORF regions of all MPXV sequences were first separated and screened. MPXV sequences before 2022 (NC_003310.1) and post 2022 (NC_063383.1) were separately selected as reference sequences to manually filter their ORF regions, which were templates for subsequent resolution of all MPXV ORFs (Figure 2A). By calculating the LD value and analyzing homology with template ORF sequences, a total of 165 ORF regions were selected to form ORF clusters for subsequent analysis. The DCR characteristics, initially introduced in our preceding research, represented a novel approach for learning the general host adaptation of viruses. This methodology has demonstrated its efficacy in analyzing evolutionary trends and predicting host adaptation in SARS-CoV-2 (Jing et al., 2022), bat coronaviruses (Jing et al., 2023), and swine coronaviruses (Shuyang et al., 2023). Consequently, machine learning models built upon DCR characteristics have proven to be highly effective in delineating the host adaptation properties of various viruses with a significant degree of precision. Then, T-SNE and PCA based on DCR characteristics were conducted for visualization and dimensional reduction of each type of compositional trait of ORF clusters sequences of MPXV. The results showed a separation among Congo Basin clade, West African clade and post 2022 MPXV clade in the two reduced t-SNE components of the 1536-dimentional-DCR (Figure 2B). The data linearity was further evaluated to reflect its continuity and distinguishability, as well as to support the machine learning classification of these samples. The linearity feature was designed as the ratio of the data range of PCA1 to the data range of PCA2 based on the orthogonal distribution between PCA1 and PCA2 (Figure 2C), showing that MPXV post 2022 possessed an obvious linear adaptation characteristic compared with ones before 2022. Due to the component 1 was the primary contributor to the variance in the data reduced by PCA, the results also showed a linear distribution of Congo Basin clade, West African clade and post 2022 MPXV clade from top to bottom of the Y-axis (PCA1). It meant that the evolution of all MPXV might follow a linear adaptation process. To further prove the linear adaptation, we have conducted machine learning analyses only on the MPXV of the West African clade and post 2022 clade (Figure 2D), as well as focusing solely on post 2022 MPXV clade (Figure 2E). The results showed that the linear fitting of the post 2022 MPAV clade showed a good correlation with an R-squared value of 0.28, indicating a positive linear relationship in its host adaptability. In order to further verify the reliability of linear adaptation characteristic, randomly sampling was taken from sequences before and post 2022, and a total of 123 sequences were obtained. The sampled sequences were also significantly separated on the basis of two main components reduced by both t-SNE (Figure 2F) and PCA (Figure 2G) in DCR characteristics. What’ more, the MPXV post 2022 was closely related to the West African clade (before 2022) and was on the same evolutionary trend line (Figures 2C, G). It indicated that the MPXV post 2022 had high homology with the West African clade and had underwent further evolution to be adaptable to human. The relationship between the sampled sequences by hierarchical clustering based on the DCR characteristics of ORF clusters was similar to the distribution obtained by reduction, showing clear discrimination (Figure 2H). These results showed a clear separation of MPXV before 2022 (divided into “Congo Basin” clade and “West African” clade) and post 2022, which also indicated linear adaptation of MPXV.

### 3.3 Phylogenetic analysis of highly homologous ORF clusters of MPXV

**FIGURE 3:**
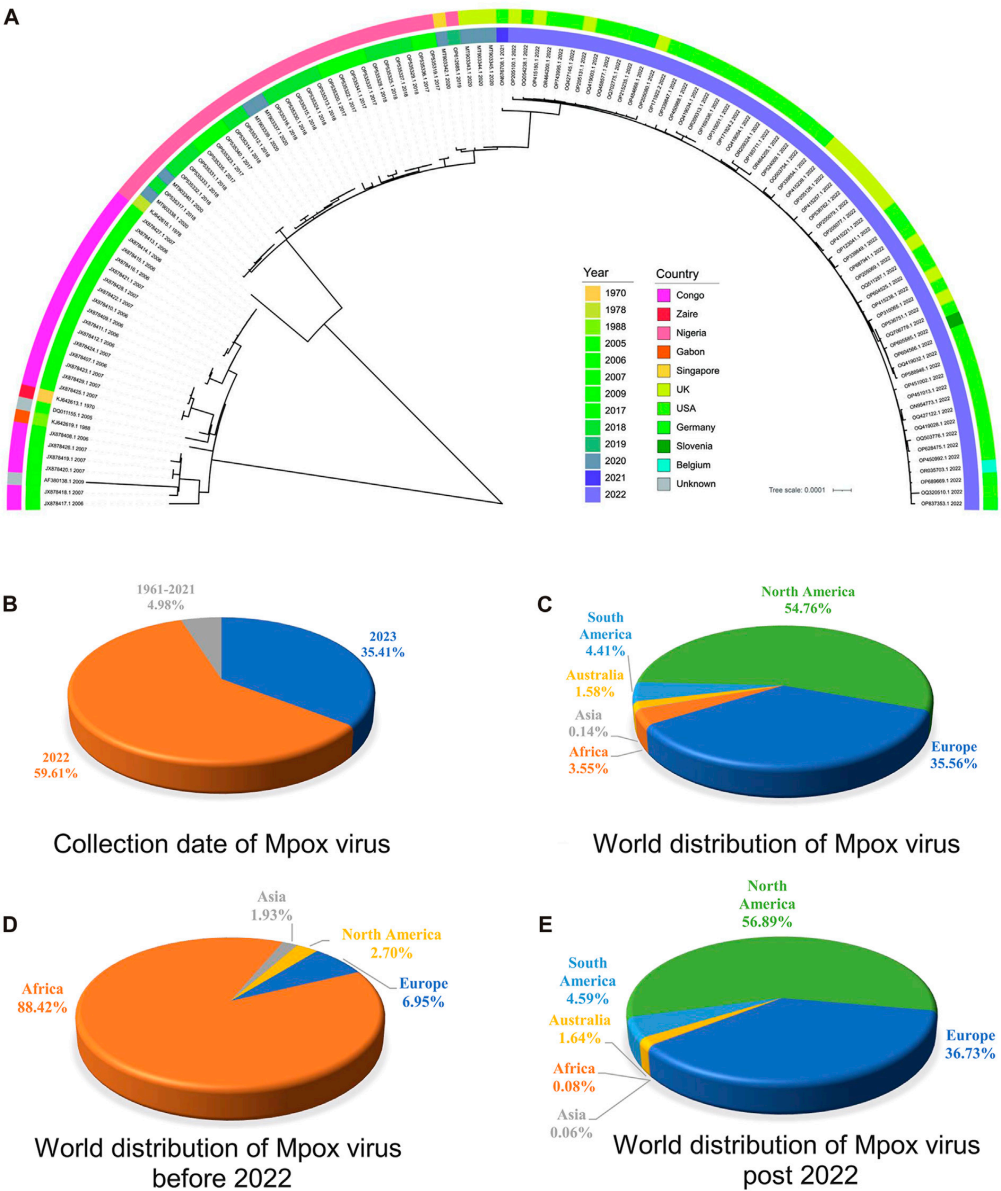
Phylogenetic analysis of highly homologous ORF clusters of MPXV. **(A)** The phylogenetic tree was constructed using iqtree with 100 bootstrap replicates for 165 ORF clusters from randomly sampled MPXV sequence. **(B)** Collection date of MPXV in public database. **(C)** World distribution of MPXV in public database. **(D)** World distribution of MPXV before 2022 in public database. **(E)** World distribution of MPXV post 2022 in public database.

Phylogenetic analysis is classical for understanding the evolutionary relationships and genetic divergence in virology for tracking virus spread and studying genetic diversity (Washburne et al., 2018). In order to elucidate the phylogenetic relationships between the MPXV sequences before and post 2022, a phylogeny tree was constructed on the basis of 165 ORF clusters of 123 sampled sequences. The phylogenetic results showed two major branches, the Congo Basin clade and the West African clade, while the West African clade was further divided into two smaller branches, the traditional West African strains (sequences before 2022) and strains post 2022 (Figure 3A). It indicated that MPXV post 2022 were more closely related to the traditional West African strains. These results were consistent with the unsupervised learning with 1536-dimentional-DCR characteristics, confirming the accuracy of the hierarchical clustering with machine learning methods. However, the phylogenetic analysis results could not directly reveal the evolutionary trends of virus. From the annotation information of phylogenetic tree, it could also be observed that the traditional Congo Basin clade and West African clade strains were mainly isolated from African countries, while MPXV strains post 2022 had spread globally (Figure 3A). Additionally, the sequences of MPXV in the public database were mainly isolated in 2022 and 2023 (accounting for 95.02%) (Figure 3B), and mainly distributed in North America (account for 54.76%) and Europe (account for 35.56%) (Figure 3C) as results of the 2022 global epidemic spread. Before 2022, the majority of MPXV was only distributed in Africa (account for 88.42%), with North America and Europe only accounting for a small percentage (2.70% & 6.95%) (Figure 3D), however, in 2022 and 2023, the proportion of cases in North America and Europe separately increased to 56.89% and 36.73%, while Africa accounted for a smaller percentage (1.64%) (Figure 3E). These results indirectly reflected the adaptive evolution of MPXV to be more adaptable to humans, consistent with the machine learning analysis.

### 3.4 Unsupervised learning of ORFs with high mutation regions

**FIGURE 4:**
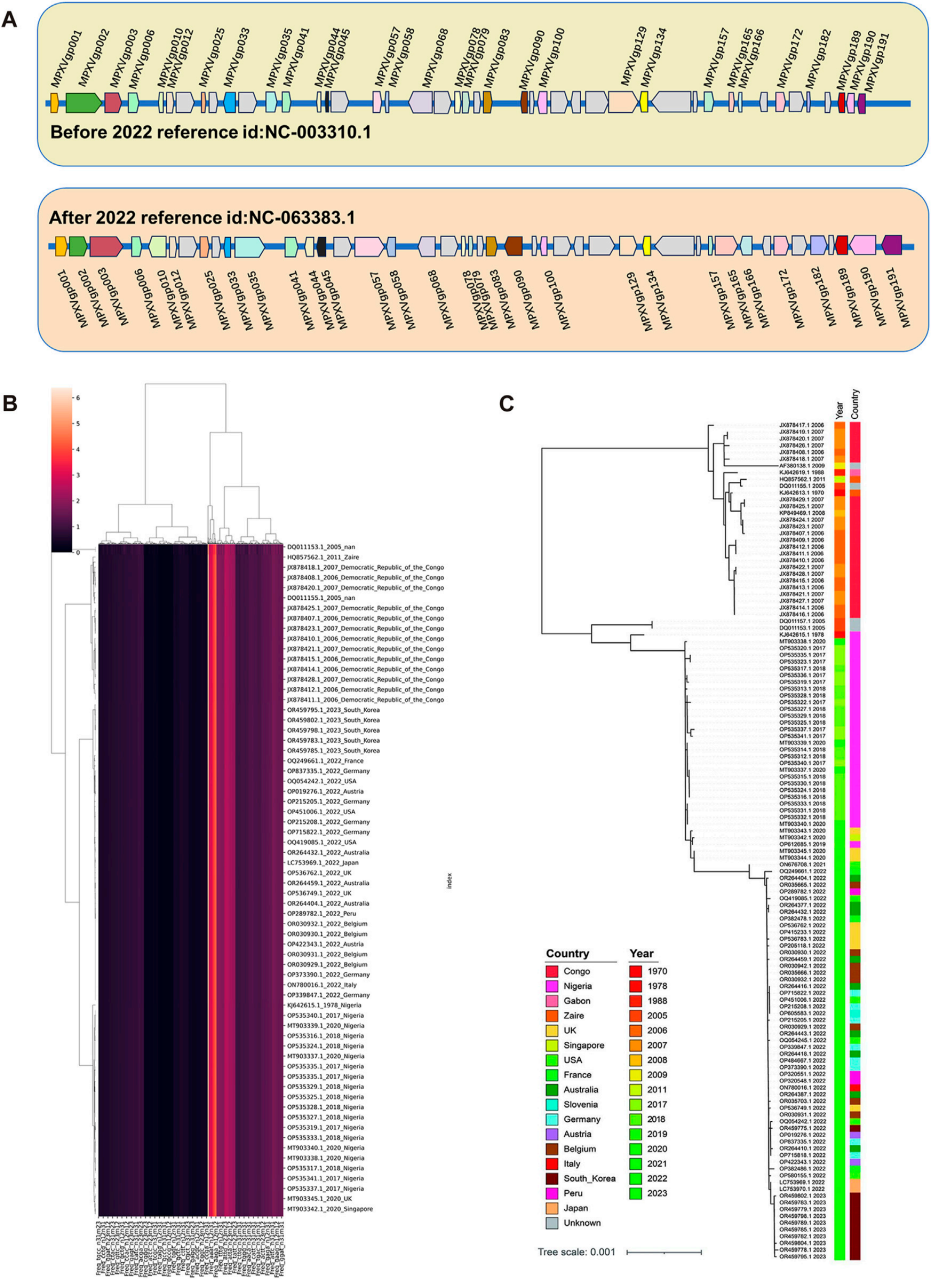
Unsupervised Learning of ORFs with high mutation regions. **(A)** The relative positions of selected 30 high mutation ORF regions in the genome of MPXV. **(B)** Hierarchical clustering of DCR characteristics of 30 high mutation ORF regions from each randomly sampled MPXV sequence. **(C)** The phylogenetic tree was constructed using iqtree with 100 bootstrap replicates for 30 high mutation ORF regions from randomly sampled MPXV sequence.

Based on the machine learning of the whole genomic composition characteristics of MPXV, it revealed the presence of linear adaptation evolution. To further screen and validate the key regions that might affect this linear adaptation, the homogeneity differences of 165 ORFs in the MPXV genome before and post 2022 were calculated based on LD values. A total of 30 major variant ORF regions were selected and the relative positions of them in the genome were shown in Figure 4A. There were 6 ORFs located in the tandem repeat regions at both ends, which were also considered to be high mutation regions, and other 24 ORFs located in the central conserved region. Subsequently, randomly sampling was taken from sequences before and post 2022, and a total of 132 sequences (67 before 2022 and 65 post 2022) were obtained. These 30 major variant ORFs of sampled sequences were analyzed by unsupervised learning. Sequences were analyzed according to DCR characteristic and then conducted by dimensionality reduction analysis, and the hierarchical clustering results showed clear discrimination between strains before and post 2022 (Figure 4B). Additionally, phylogenetic analysis was conducted on 30 major variant ORFs of sampled sequences, and the results showed three main branches, Congo Basin clade, West African clade and post 2022 MPXV clade, and the post 2022 MPXV clade was more closely related to the West African clade (Figure 4C), which were consistent with the hierarchical clustering results and the full-length 165 ORF clusters phylogenetic analysis results. These indicated that the disparities within the main mutant domains of the MPXV were pivotal in shaping the linear adaptive evolutionary trend of the genomic landscape.

### 3.5 The structure prediction of key mutant proteins by AlphaFold 2

**FIGURE 5:**
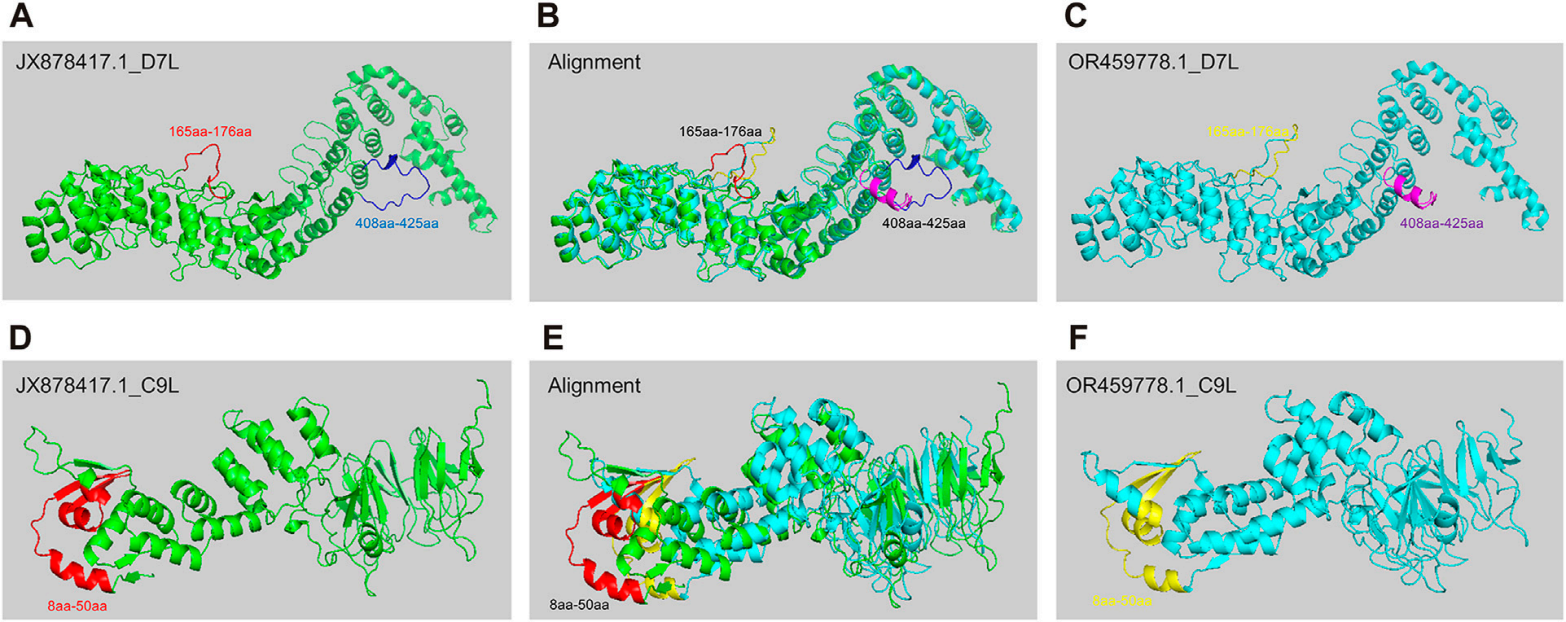
The structure prediction of key mutant proteins by AlphaFold 2. **(A–C)** Structure prediction results of D7L protein of MPXV JX878417.1 strain **(A)** and OR459778.1 strain **(C)**, and alignment of two structures **(B)**. **(D–F)** Structure prediction results of C9L protein of MPXV JX878417.1 strain **(D)** and OR459778.1 strain **(F)**, and alignment of two structures **(E)**.

Predicting protein structure is important for understanding pathogen evolution, by which researchers can gain insights into how these proteins evolve or interact with hosts. AlphaFold 2, developed by DeepMind, is a deep learning system that accurately predicts the 3D structure of a protein based on its amino acid sequence, leveraging evolutionary information and multiple sequence alignment to generate highly accurate protein structure predictions (Senior et al., 2020). To study the potential differences in protein structure changes during the evolution of MPXV, we selected two strains with significant differences as representative strains before and post 2022 (JX878417.1 & OR459778.1) for protein structure prediction based on hierarchical clustering and phylogenetic analysis results. Later, based on the distribution of SNP sites in 30 ORF regions and the functions of encoded protein, D7L (Yanjiao et al., 2023) and C9L (UKHSA, 2022)proteins, which might be associated with virus replication and immune evasion, were selected for structural prediction. The results indicated that D7L protein formed two different coil structures between amino acids 165-176 in the two MPXV strains (Figures 5A–C). The coil structure could provide binding sites between proteins, thereby participating in biological processes such as protein interactions and signal transduction (Nicholas et al., 2017). Additionally, between amino acids 408-425, the JX878417.1 strain exhibited a coil structure (Figures 5A, B), whereas the OR459778.1 strain primarily consisted of an α-helix (Figures 5B, C). For the C9L protein, the overall structural differences between the two strains were greater compared to D7L (Figures 5B, E), mainly reflected in the N-terminus between amino acids 8-50. Although both strains exhibited alternating forms of α-helix, coil, and β-sheet in this region, there were obvious structural differences between amino acids 8-50 (Figures 5D–F). However, in the alignment of these protein sequences, in addition to the main structurally different sites mentioned above, there were also other single amino acid differing sites, but these differences did not manifest as significant structural variances. This meant that sequence differences might not necessarily absolutely affect protein structure. These results indicated that as MPXV spread in humans, the protein associated with pathogenicity could also be affected by structural variations, which might be one of the internal reasons for the linear adaptive evolution of MPXV and deserved further study.

## 4 Discussion

Key data from our study illustrated a clear linear adaptive evolution in MPXV sequences post 2022, suggesting an increased adaptation to the human host due to accumulated mutations. This study offers valuable insights into the evolutionary patterns of MPXV in 2022 through genomic composition characteristic analysis using machine learning methods.

Biological sequences, such as genome sequences, typically exhibit advantageous textual characteristics conducive to analysis. The essential information concealed within the original sequence data can be revealed by numerically transforming and characterizing the sequence information, followed by applying machine learning or deep learning techniques (Angermueller et al., 2016; Brian et al., 2021). Unsupervised learning methods have achieved excellent representation effects for protein (Zhen et al., 2018), DNA (Laiyi et al., 2020), and RNA (Xiaoyong et al., 2018) sequences. The genomic compositional analysis method is a genome sequence representation method that does not rely on pre-training and boasts fast computational speed. It utilizes the differences in genomic composition between different organisms to represent gene characteristics in a compositional numerical space, demonstrating good linearity, which can be used for machine learning research. DCR characteristics is a genomic compositional representation method that we previously proposed, which has shown good representation effects for the evolution and host adaptability of viruses (Jing et al., 2022; Jing et al., 2023; Shuyang et al., 2023). Machine learning based on DCR characteristics analysis of the MPXV genome will help to better explain the reasons for its outbreak in 2022.

Here, we focused mainly on the complex genomic information analysis of the monkeypox virus, and described its evolutionary trends based on genomic composition characteristics using machine learning. Previous studies on the genomic sequences of MPXV have mainly relied on classical homology comparisons and phylogenetic analysis (Hongling et al., 2023; Marie et al., 2023). Due to the complexity of the MPXV genome, the ORF regions were first cleaned and analyzed to delete ones with poor homology, which would cause significant bias in the machine learning results. Unsupervised machine learning on 165 highly homologous ORF clusters based on DCR characteristics demonstrated a clear linear adaptability of the evolutionary trend of MPXV. Linear adaptation is a process in which viruses evolve in a consistent and predictable manner over time (Sanjuán and Domingo-Calap, 2016; Primadharsini et al., 2021). This type of evolution occurs when the virus undergoes gradual changes in response to selective pressures, such as the host’s immune system or antiviral drugs (Duggal and Emerman, 2012). As a result, the virus may develop mutations that allow it to persist and replicate more effectively within the host (Juan et al., 2014; Sun et al., 2014). The linear adaptive evolution of MPXV might be reflected in its continuous expansion of the geographical spread, more pronounced human-to-human transmission characteristics, and improved adaptation to human hosts, which were obvious features of MPXV post 2022 (Hatmal et al., 2022; Thornhill et al., 2022). These explained the outbreak of monkeypox in 2022 from the perspective of genomic composition characteristics. Recent studies have shown that MPXV might have been circulating and evolving within human populations since 2016 to evade the human immune system (Áine et al., 2023). Meanwhile, researchers found that the accelerated evolution MPXV was potentially driven by the action of host APOBEC3 enzymes, as the mutations follow signatures of APOBEC3-mediated editing. Early signs of microevolution were seen, with 15 additional SNPs emerging within the outbreak cluster, also following the APOBEC3 mutation bias. Ongoing viral intra-patient diversity and minor variants were observed, again with an APOBEC3 signature, in some cases targeting immune-related viral genes (Isidro et al., 2022). It explained the potential mechanisms for the adaptive evolution of the MPXV at the level of the host immune system. This was consistent with our linear adaptation conclusion, the MPXV gradually gained adaptive advantages during its long-term evolution and continuous interactions with human hosts, leading to a sudden outbreak. What’s more, the global spread of the MPXV might further exacerbate its trend of adaptive evolution.

The mutation rate of MPXV is lower than RNA viruses. Estimated through molecular clock analysis, the nucleotide substitution rate of MPXV ranges from 2 × 10^−6^ to 1 × 10^−5^ (nucleotide substitutions/site/year), which is 1-2 orders of magnitude lower than RNA viruses (Xiang and White, 2022). However, the 2022 MPXV differs from the related 2018-2019 viruses by an average of 50 SNPs, which is substantially higher (approximately 6–12 times more) than anticipated based on previous estimates of the substitution rate for Orthopoxviruses (Isidro et al., 2022). Our study screened 30 ORFs related to linear adaptation of MPXV. Unsupervised learning results confirmed that the dimensionality reduction analysis of these 30 ORFs sequences also showed significant linear evolution. Furthermore, comparison revealed that these 30 ORF regions were largely consistent with the previous study where the high-mutation SNP sites located (Isidro et al., 2022). Accurate protein structure prediction could play significant roles in advancing understanding of pathogen evolution (Guangyu et al., 2023). The subsequent predicted results of the major mutant protein structures showed obvious differences in the protein structures of MPXV before and post 2022. Variations in the three-dimensional arrangement of proteins can alter their biological activity and interactions with other molecules, which could potentially further affect the virus’s host adaptability and transmission capabilities. These highly mutable ORF regions (SNP sites) might affect the protein function by altering the structure of key domains, ultimately leading to the linear adaptive evolution of MPXV.

However, research like this article, which relies on a public MPXV sequence database, is influenced by the quality and distribution of the available sequence data. The results of this study were somewhat affected by the uneven distribution of samples before and post 2022, in which the number of samples before 2022 was small. We reduced the error by using random sampling to make the sample sizes more consistent. What’s more, the unsupervised learning data analysis method we used could make inferences about the evolution direction of MPXV, nevertheless, the single data label prevents us from conducting supervised learning, thus hindering the prediction of the adaptability of MPXV to humans. This also constitutes the research direction we are presently endeavoring to pursue.

In summary, the machine learning results of 165 ORF clusters based on DCR characteristics indicated that MPXV sequences post-2022 showed a clear linear adaptive evolution, suggesting an increased adaptation to the human host due to accumulated mutations. To enhance accuracy, the ORF regions with significant variations were excluded based on homogeneity difference, narrowing down the key ORF clusters and reinforcing the conclusion of linear adaptability. Subsequently, AlphaFold 2 was employed to predict key differential protein structures, suggesting that differences in main domains could be a contributing factor to the observed linear adaptive evolution. Linear adaptation is a key factor in the ongoing arms race between viruses and their hosts, and understanding this process is crucial for developing effective strategies to combat viral infections. Consequently, this study offers valuable insights into the evolutionary patterns of MPXV in 2022 through genomic composition characteristic analysis using machine learning methods.

## Data Availability

The datasets presented in this study can be found in online repositories. The names of the repository/repositories and accession number(s) can be found in the article/Supplementary material.
